# Sera of patients infected by earlier lineages of SARS-CoV-2 are capable to neutralize later emerged variants of concern

**DOI:** 10.1093/biomethods/bpac021

**Published:** 2022-08-22

**Authors:** Alex Pauvolid-Corrêa, Braulia Costa Caetano, Ana Beatriz Machado, Mia Araújo Ferreira, Natalia Valente, Thayssa Keren Neves, Kim Geraldo, Fernando Motta, Valdiléa Gonçalves Veloso dos Santos, Beatriz Grinsztejn, Marilda Mendonça Siqueira, Paola Cristina Resende

**Affiliations:** Laboratório de Vírus Respiratórios e Sarampo, COVID-19 National Reference Laboratory of Brazil and World Health Organization COVID-19 Reference Laboratory, Instituto Oswaldo Cruz, Fundação Oswaldo Cruz (Fiocruz), Rio de Janeiro, Brazil; Department of Veterinary Integrative Biosciences, Texas A&M University, College Station, Texas, United States of America; Departamento de Veterinária da Universidade, Federal de Viçosa (UFV), Viçosa, Brazil; Laboratório de Vírus Respiratórios e Sarampo, COVID-19 National Reference Laboratory of Brazil and World Health Organization COVID-19 Reference Laboratory, Instituto Oswaldo Cruz, Fundação Oswaldo Cruz (Fiocruz), Rio de Janeiro, Brazil; Laboratório de Vírus Respiratórios e Sarampo, COVID-19 National Reference Laboratory of Brazil and World Health Organization COVID-19 Reference Laboratory, Instituto Oswaldo Cruz, Fundação Oswaldo Cruz (Fiocruz), Rio de Janeiro, Brazil; Laboratório de Vírus Respiratórios e Sarampo, COVID-19 National Reference Laboratory of Brazil and World Health Organization COVID-19 Reference Laboratory, Instituto Oswaldo Cruz, Fundação Oswaldo Cruz (Fiocruz), Rio de Janeiro, Brazil; Laboratório de Vírus Respiratórios e Sarampo, COVID-19 National Reference Laboratory of Brazil and World Health Organization COVID-19 Reference Laboratory, Instituto Oswaldo Cruz, Fundação Oswaldo Cruz (Fiocruz), Rio de Janeiro, Brazil; Laboratório de Vírus Respiratórios e Sarampo, COVID-19 National Reference Laboratory of Brazil and World Health Organization COVID-19 Reference Laboratory, Instituto Oswaldo Cruz, Fundação Oswaldo Cruz (Fiocruz), Rio de Janeiro, Brazil; Instituto Nacional de Infectologia Evandro Chagas (INI), Fiocruz, Rio de Janeiro, Brazil; Laboratório de Vírus Respiratórios e Sarampo, COVID-19 National Reference Laboratory of Brazil and World Health Organization COVID-19 Reference Laboratory, Instituto Oswaldo Cruz, Fundação Oswaldo Cruz (Fiocruz), Rio de Janeiro, Brazil; Instituto Nacional de Infectologia Evandro Chagas (INI), Fiocruz, Rio de Janeiro, Brazil; Instituto Nacional de Infectologia Evandro Chagas (INI), Fiocruz, Rio de Janeiro, Brazil; Laboratório de Vírus Respiratórios e Sarampo, COVID-19 National Reference Laboratory of Brazil and World Health Organization COVID-19 Reference Laboratory, Instituto Oswaldo Cruz, Fundação Oswaldo Cruz (Fiocruz), Rio de Janeiro, Brazil; Laboratório de Vírus Respiratórios e Sarampo, COVID-19 National Reference Laboratory of Brazil and World Health Organization COVID-19 Reference Laboratory, Instituto Oswaldo Cruz, Fundação Oswaldo Cruz (Fiocruz), Rio de Janeiro, Brazil

## Abstract

Serum samples of 20 hospitalized COVID-19 patients from Brazil who were infected by the earlier SARS-CoV-2 lineages B.1.1.28 and B.1.1.33, and by the variant of concern (VOC) Gamma (P.1) were tested by plaque reduction neutralization test (PRNT_90_) with wild isolates of a panel of SARS-CoV-2 lineages, including B.1, Zeta, N.10, and the VOCs Gamma, Alpha, and Delta that emerged in different timeframes of the pandemic. The main objective of the present study was to evaluate if serum of patients infected by earlier lineages was capable to neutralize later emerged VOCs. We also evaluated if the four-fold difference in PRNT_90_ titers is a reliable seropositivity criterion to distinguish infections caused by different SARS-CoV-2 lineages. Sera collected between May 2020 and August 2021 from the day of admittance to the hospital to 21 days after diagnostic of patients infected by the two earlier lineages B.1.1.28 and B.1.1.33 presented neutralizing capacity for all challenged VOCs, including Gamma and Delta. Among all variants tested, Delta and N.10 presented the lowest geometric mean of neutralizing antibody titers, and B.1.1.7, presented the highest titers. Four patients infected with Gamma, that emerged in December 2020, presented neutralizing antibodies for B.1, B.1.1.33 and B.1.1.28, its ancestor lineage. All of them had neutralizing antibodies under the level of detection for the VOC Delta. Patients infected by B.1.1.28 presented very similar geometric mean of neutralizing antibody titers for both B.1.1.33 and B.1.1.28. Findings presented here indicate that most patients infected in early stages of COVID-19 pandemic presented neutralizing antibodies capable to neutralize wild types of all later emerged VOCs in Brazil, and that the four-fold difference in PRNT_90_ titers is not reliable to distinguish humoral response among different SARS-CoV-2 lineages.

## Introduction

The ongoing pandemic of coronavirus disease 2019 (COVID-19) is a major healthcare threat worldwide. While viral RNA-based testing for acute infection of severe acute respiratory syndrome coronavirus 2 (SARS-CoV-2) is the current standard, surveying antibodies is important to determine the past exposure (Muruato et al., 2021). Despite the relationship between humoral response and clinical protection from SARS-CoV-2 infection remains not fully understood, some studies have confirmed neutralizing antibodies as an immune correlate of protection (Khoury et al., 2021). The humoral immune response can block infection through neutralizing antibodies, which bind the virus in a manner that prevents host cell infection (VanBlargan et al., 2016). The host humoral response against SARS-CoV-2, including IgA, IgM, and IgG response, has been examined mostly by ELISA-based assays using recombinant viral nucleocapsid protein or pseudovirus-based neutralization assays (Tang et al., 2019). Coronavirus infections typically induce neutralizing antibody responses, and virus neutralization assays performed on cell cultures, as plaque reduction neutralization test (PRNT), are considered as gold standard for serological testing and determining immune protection (Muruato et al., 2021). Although antiviral T cell certainly contribute some degree of protection, strong evidence of a protective role for neutralizing serum antibodies synthetized by B cell memory exists (Le Bert et al., 2020). Neutralizing antibody levels are highly predictive of immune protection from symptomatic SARS-CoV-2 infection (Khoury et al., 2021).

Since the pandemic began in China in December 2019, thousands of SARS-CoV-2 lineages have emerged worldwide (Koyama et al., 2020). The variants that presented increased transmissibility, virulence, and decreased response to available diagnostics, vaccines, and therapeutics were defined by the World Health Organization as variant of concern (VOC) (Konings et al., 2021). Following the upsurge of variants, several reinfection cases started to be reported worldwide raising questions about the efficiency of humoral response mounted after primary infections to prevent a secondary infection by SARS-CoV-2 (Babiker et al., 2021). In fact, some recent studies have experimentally demonstrated that monoclonal antibodies, convalescent plasma of individuals infected by earlier lineages, and serum collected from vaccinees have a reduced neutralizing capacity when challenged with the recently emerged VOCs of SARS-CoV-2 (Dejnirattisai et al., 2021b; Liu et al., 2021; Vidal et al., 2021).

The cross-reactivity of humoral responses among all different lineages of SARS-CoV-2 including VOCs, as well as the potential use of specific serological methods as PRNT to distinguish lineage infections remain unclear. For instance, monotypic reactions or four-fold difference in PRNT titers is commonly used to distinguish exposure to closely related flaviviruses and its serotypes (Pauvolid-Corrêa et al. 2014). If PRNT titers can distinguish exposure to different SARS-CoV-2 lineages remains unknown. Several lineages of SARS-CoV-2 have been reported in Brazil by the consortium COVID-19 Fiocruz Genomics Surveillance Network of the Brazilian Ministry of Health (http://www.genomahcov.fiocruz.br/dashboard/). Among the most important lineages detected in the country were the VOCs Gamma (also referred as P.1), Delta (B.1.617), Alpha (B.1.1.7), and Omicron (B.1.1.529) as well as the Zeta (P.2), B.1, B.1.1.28, B.1.1.33 and N.10. The main objectives of the present study were to assess the capacity of sera of COVID-19 patients infected by earlier lineages of SARS-CoV-2 to neutralize later emerged VOCs, and also to evaluate if the $PRNT_{90}$ is a reliable serologic method to distinguish infections caused by different SARS-CoV-2 lineages.

## Methods

*Case description*: Swab and serum samples were periodically collected from over 70 hospitalized COVID-19 patients that were admitted between May 2020 and August 2021 to the COVID-19 Hospital of the National Institute of Infectious Diseases, Rio de Janeiro, Brazil. Samples were weekly collected from the day of admittance up to 21 days after diagnostic. The index cases were patients aged 18 years and older with no history of vaccination at the time of sampling. All patients had SARS-CoV-2 infection confirmed by real-time reverse transcription polymerase chain reaction (qRT-PCR) in respiratory samples, which consisted of a combination of two nasopharyngeal swabs and one oropharyngeal swab collected in 3 mL of viral transportation medium (VTM).

*RT-qPCR for SARS-CoV-2*: RNA was extracted from respiratory samples using Chemagic Viral DNA/RNA 300 kit H96 in a Janus G3 automated workstation (Perkin Elmer, www.chemagen.com). SARS-CoV-2 was detected by RT-qPCR assays targeting the viral gene E. Reactions were performed using the Kit Molecular SARS-CoV-2 (E/RP) (Bio-Manguinhos/IBMP, Brazil).

*Whole-genome sequencing*: Positive samples eligible for whole-genome sequencing had RNA extracted using the QIAamp Viral RNA Mini Kit (QIAGEN) or using Chemagic Viral DNA/RNA 300 kit H96 in a Janus G3 automated workstation (Perkin Elmer, www.chemagen.com). The SARS-CoV-2 genomes were recovered by amplification of long segments (2kb), according to a protocol developed by COVID-19 Fiocruz Genomic Surveillance Network to recover high-quality genomes (P.C. Resende, unpub. data, doi.org/10.1101/2020.04.30.069039). Segment libraries were then sequenced in Illumina MiSeq. The FASTQ reads obtained were imported into the CLC Genomics Workbench version 20.0.4 (QIAGEN), trimmed, and mapped against the reference sequence hCoV-19/Wuhan/WIV04/2019 (GISAID access number EPI_ISL_402124) to obtain the final genome consensus. The SARS-CoV-2 lineage characterization was performed by Pango Network (O'Toole et al., 2021). All genomic and epidemiological data associated were uploaded at the EpiCoV database in the GISAID (www.gisaid.org).

*Isolation of SARS-CoV-2 reference lineages used for PRNT*: As part of the Brazil's Surveillance Network for Respiratory Illnesses, respiratory samples collected in sentinel units located in different states of the country are routinely sent to the Reference Laboratory. Eligible samples that tested positive for different lineages of SARS-CoV-2 were submitted to virus isolation in Vero E6 or Vero CCL-81 cells at a Biosafety level 3 Laboratory, as previously described (Basile et al., 2020). Briefly, 200 µL of each respiratory sample were inoculated in cell cultures, which were then inspected daily for cytopathic effect (CPE), up to four days. For each sample, isolation was attempted in a maximum of three consecutive blind passages. Overall, in positive cultures, CPE started on second day post-infection and viral harvest was performed at the fourth day post-infection. When CPE was observed, culture supernatants were aliquoted in working stocks and an aliquot submitted to RT-qPCR followed by nucleotide sequencing for lineage confirmation. Once confirmed, the consensus sequences were deposited at the EpiCoV data base on GISAID (www.gisaid.org), and one representative of each SARS-CoV-2 lineage sequentially titrated by lysis plaque assay, to form a lineage bank with reference isolates.

*PRNT*: Subsets of serum samples of hospitalized patients that were positive for different lineages of SARS-CoV-2 were selected for PRNT. For the patients who had several blood samples collected during hospitalization, the last samples were preferred. Sera were heat-inactivated at 56°C for 30 minutes to inactivate the complement system. Heat-inactivated serum samples were subjected to $PRNT_{90}$ in Vero cells (ATCC, CCL 81) maintained in cell culture medium supplemented with fetal bovine serum, sodium bicarbonate, antibiotics and incubated in 5% $CO_2$ atmosphere at 37℃. Briefly, inactivated aliquots were screened at a single dilution of 1:10 in two to three-day old Vero CCL-81 cells seeded in 6-well plates. Exceptionally, samples with low volume were screened at 1:20. Samples that were reactive for SARS-CoV-2 were then tested in duplicates in serial two-fold dilutions that ranged from 1:10 to up to 1:320 for their ability to neutralize 50-80 plaques forming units (PFU) by each one of five infectious SARS-CoV-2 reference lineages isolates. The panel of reference isolates used for PRNT included the lineages B.1 (hCoV-19/Brazil/RJ-FIOCRUZ-314/2020, GISAID accession number EPI_ISL_414045), B.1.1.28 (hCoV-19/Brazil/AL-FIOCRUZ-33444-1P/2020, EPI_ISL_2645638), B.1.1.33 (hCoV-19/Brazil/RJ-FIOCRUZ-20136-1P/2020, EPI_ISL_1181430), Zeta (hCoV-19/Brazil/PB-FIOCRUZ-33096-1P/2020, EPI_ISL_1402429), N.10 (hCoV-19/Brazil/MA-FIOCRUZ-6871-1P/2021, EPI_ISL_3828018), and the VOCs Gamma (hCoV-19/Brazil/AM-FIOCRUZ-3521-1P/2021, EPI_ISL_1402431), Alpha (hCoV-19/Brazil/RJ-FIOCRUZ-2624-1P/2021, EPI_ISL_1402430) and Delta (hCoV-19/Brazil/MA-FIOCRUZ-25688-2P/2021, EPI_ISL_2645417). After 48h of incubation, plates were overlaid with neutral red solution, and after 72h, PFUs were visualized and counted through a transilluminator. Serum samples were considered reactive to SARS-CoV-2 when a serum dilution of at least 1:10 reduced no less than 90% of the PFU of SARS-CoV-2, as previously reported (Deshpande et al., 2020). Serum samples that presented $PRNT_{90}$ titers ≥320 and had enough volume available were retested in higher dilutions to reach endpoint titers. Serum samples were considered seropositive to a specific lineage of SARS-CoV-2 when it had $PRNT_{90}$ titer of at least 10 and were seronegative for all other lineages in monotypic reactions. Additionally, samples that were reactive for a lineage and its reciprocal neutralizing antibody titer was at least four-fold greater than what was observed for the other tested lineage were also considered seropositive in heterologous reactions. Specific neutralizing antibodies were titred by $PRNT_{90}$ and the geometric mean was calculated for comparative analysis among the different lineages of SARS-CoV-2.

**Statistical analysis**

In each group of patients, the neutralizing antibody titers detected against the originally infecting SARS-CoV-2 lineage were compared to antibody titers elicited to other lineages by means of two-tailed paired t-tests, at 95% confidence level. Analysis was performed in GraphPad Prism 9.4.1 (GraphPad Software, LLC, San Diego, CA, USA).

**Ethics**

This study was approved by the Ethics Committee of the Fundação Oswaldo Cruz (CAAE 68118417.6.0000.5248), and the Ethics Committee of the National Institute of Infectious Diseases (CAAE 32449420.4.1001.5262). All patients entering the study were required to read and sign an informed consent form.

**Results**

From a population of patients recruited from May 2020 to August 2021, we were able to obtain a panel of 20 sera of individuals infected by SARS-CoV-2 lineages B.1.1.28, B.1.1.33, the two most common lineages in early pandemic (Resende et al., 2021) and Gamma, the first VOC to become prevalent in Brazil (Naveca et al., 2021). All 20 patients had detectable $PRNT_{90}$ titers for at least one of the eight lineages tested. Of these 20 serum samples, five were from patients positive for B.1.1.28, four from patients that tested positive for Gamma, and 11 were positive for B.1.1.33 (Table). Among five patients infected by B.1.1.28, highest neutralizing antibody titers were observed for B.1.1.28, followed by Alpha, B.1.1.33, B.1, Gamma, Zeta, N.10 and Delta (Figure). Among patients infected by B.1.1.33, similar profile was observed. Neutralizing antibody titer geometric mean was higher for B.1.1.33, followed by Alpha, B.1, B.1.1.28, Zeta, Gamma, and with equally reduced neutralizing antibodies for Delta and N.10 (Figure). From four patients infected by Gamma, highest $PRNT_{90}$ titers were observed for Gamma, followed by Alpha, B.1.1.33, N.10, B.1.1.28 and Zeta, and with neutralizing antibodies under the level of detection for Delta in all individuals (Table).

The four-fold difference among PRNT titers was not a suitable to distinguish infections among the different lineages of SARS-CoV-2 tested. Convalescent serum from patients infected by B.1.1.28 presented a geometric mean of PRNT titer very similar for B.1.1.28, B.1.1.33 and Alpha lineages, and the titer difference was less than four-fold for B.1, even though the anti-B.1 antibody titer levels were significantly lower ($p$=0,02). In fact, $PRNT_{90}$ titers for B.1.1.28 in patients infected by the same virus were not even two-fold greater when compared to B.1.1.33 or the VOC Alpha. Four-fold difference for B.1.1.28 was observed only when compared to the titers of the variants N.10, Zeta and Delta (Table). A similar profile was observed for samples from patients infected by B.1.1.33. In this case, these patients presented similar PRNT titers between B.1.1.33, B.1 and Alpha. The geometric mean of antibody titer in B.1.1.33-induced sera was less than four-fold greater for B.1.1.33 when compared to B.1.1.28, Zeta and N.10. Four-fold or greater B.1.1.33 titers were observed only when compared to Gamma and Delta VOCs. Finally, for the patients previously infected by Gamma, PRNT titers were more than four-fold higher for Gamma when compared to B.1 and B.1.1.28, Delta and Zeta. Differences were smaller than four-fold when compared to B.1.1.33, N.10, and Alpha (Table).

Table: $PRNT_{90}$ titers of convalescent sera of COVID-19 patients for different SARS-CoV-2 lineages

| *Patient ID* | *Lineage of infection* | *SARS-CoV-2 lineages* | | | | | | | |
|---|---|---|---|---|---|---|---|---|---|
| | | *B.1* | *B.1.1.28* | *B.1.1.33* | *N.10* | *Zeta* | *Alpha* | *Gamma* | *Delta* |
| COV029 | B.1.1.28 | 80 | 160 | 160 | 40 | 80 | 80 | 80 | 40 |
| COV161 | B.1.1.28 | 40 | 160 | 160 | 10 | 10 | 80 | 20 | 10 |
| COV168 | B.1.1.28 | 80 | 320 | 320 | 20 | 20 | ≥320 | 40 | 10 |
| COV167 | B.1.1.28 | 160 | 320 | 160 | 20 | 20 | ≥320 | 10 | 40 |
| COV178 | B.1.1.28 | 40 | 80 | 80 | 40 | 40 | 80 | 40 | <10 |
| **Geometric Mean<br>(95% CI)** | **B.1.1.28** | **69.6<br>(33.9 – 143.1)** | **183.8<br>(89.4 – 377.6)** | **160<br>(87 – 294)** | **22.9<br>(11.2 – 47.2)** | **26.4<br>(9.9 – 70.4)** | **160<br>(67.7 – 378.4)** | **30.3<br>(11.4 – 80.9)** | **10.9<br>(1.7 – 71.5)** |
| COV014 | B.1.1.33 | 20 | 40 | 160 | <10 | 20 | 40 | 10 | <20 |
| COV057 | B.1.1.33 | 160 | 80 | 80 | 40 | 40 | 80 | 80 | NT |
| COV008 | B.1.1.33 | 160 | 80 | 160 | 80 | 80 | 160 | 80 | 20 |
| COV051 | B.1.1.33 | 40 | 40 | 160 | 20 | 40 | 160 | 80 | <10 |
| COV170 | B.1.1.33 | 160 | 160 | 160 | 40 | 40 | 80 | 40 | 20 |
| COV186 | B.1.1.33 | 40 | 80 | 160 | 20 | 20 | 80 | 40 | 40 |
| COV036 | B.1.1.33 | 640 | 80 | 640 | 40 | 160 | 640 | 80 | 40 |
| COV101 | B.1.1.33 | 320 | 160 | 160 | 80 | 160 | 320 | 160 | 40 |
| COV106 | B.1.1.33 | 40 | 160 | 160 | 20 | 40 | 80 | 40 | <10 |
| COV150 | B.1.1.33 | 80 | 320 | 160 | 10 | 20 | 80 | 20 | 10 |
| COV146 | B.1.1.33 | 320 | 160 | 320 | 160 | 160 | 320 | 80 | ≥320 |
| **Geometric Mean<br>(95% CI)** | **B.1.1.33** | **109.6<br>(52.7 – 228)** | **102.9<br>(66.9 – 158.3)** | **181.5<br>(127.9 – 257.4)** | **26.9<br>(10.9 – 66.3)** | **51.5<br>(29.3 – 90.2)** | **132.4<br>(76.1 – 230.6)** | **51.5<br>(30.5 – 86.7)** | **13.3<br>(3.1 – 49.8)** |
| COV381 | Gamma | 10 | 80 | 80 | 80 | 40 | 80 | 160 | <10 |
| COV373 | Gamma | 40 | 40 | 40 | 80 | 80 | 80 | 160 | <10 |
| COV385 | Gamma | 20 | 40 | 40 | 40 | 40 | 160 | 320 | <20 |
| COV386 | Gamma | 20 | 40 | 160 | 10 | 40 | 160 | 160 | <10 |
| **Geometric Mean<br>(95% CI)** | **P.1** | **20<br>(8.1 – 49.2)** | **47.6<br>(27.4 – 82.5)** | **67.2<br>(23.4 – 193.4)** | **40<br>(8.4 – 190.3)** | **47.5<br>(27.4 – 82.5)** | **113.1<br>(59.8 – 213.9)** | **190.3<br>(109.6 – 330.6)** | **1** |

NT: Not tested

For geometric mean calculation purposes, a value of 1 was assigned to titers <10 and <20, and a value of 320 was assigned to titers ≥320.

## Discussion

Besides being considered markers of immune protection, specific neutralizing antibodies have also been used to evaluate viral exposure, and ultimately, for diagnostic purposes. Assays designed to detect neutralization antibodies have been widely used for the diagnostic of different viral groups. The relationship between humoral response to SARS-CoV-2 antigens, especially the spike protein, and clinical protection from COVID-19 remains not fully understood, although some studies have confirmed neutralizing antibodies as an immune correlate of protection (Khoury et al., 2021). Likewise, it is still not completely understood to what extent the mutations in viral antigens contribute to virus evasion from neutralizing antibodies, and how significant this escape mechanism could be for the general effectiveness of the protective response, especially in the context of vaccinations, reinfections, and the evolution of the pandemic. It has been shown that mutations in the ACE2 binding site of variants result in an increased affinity for the receptor ACE2, and that changes outside the receptor-binding domain also impact neutralization (Dejnirattisai et al., 2021a). In this scenario, we primarily investigated the neutralization profile of sera from patients infected by the most prevalent lineages in early pandemic in Brazil including B.1.1.33 and B.1.1.28, with three later emerged VOCs including Gamma, Alpha, and Delta. Additionally, we investigated if Gamma-induced antibodies were capable to neutralize the early isolates and the VOC, Delta. We observed that, despite the variation in antibody levels, most serum samples of patients infected by early isolates presented some level of neutralizing activity against all VOCs. The reverse situation, in which serum samples from individuals infected with the Gamma lineage were challenged with early pandemic viral isolates, also produced detectable neutralization. However, when sera from Gamma-infected individuals were tested with other VOC Delta, the $PRNT_{90}$ titer was below the limit of detection of the assay (Table).

The variant Gamma, which emerged from the B.1.1.28 lineage, contains 17 amino acid substitutions, ten of which are in the spike protein, including N501Y, E484K and K417T in the receptor-binding domain, five in the N-terminal domain, and the mutation H655Y near the furin cleavage site (Wang et al., 2021). These mutations reduce the neutralization capacity of convalescent sera from individuals infected by early isolates (Dejnirattisai et al., 2021b), and findings presented here suggest these mutations perhaps interfere also with the efficiency of its neutralizing antibodies for other variants, as Delta.

Despite the small number of patients evaluated, the findings that Gamma-induced immune sera presented low or absent $PRNT_{90}$ titers for Delta raises concern over the potential risk of reinfections, and consequent prolongation of the pandemic. It is important to mention that the protective role of neutralizing antibodies does not seem to be directly linked to the titers in which they are found, but to timing and kinetics of their production, with studies suggesting a limited role of antibodies in predicting disease severity of the COVID-19, and that the earlier the presence of neutralizing antibodies after infection, the less severe is the disease outcome (Ren et al., 2020). It is noteworthy that for most viruses there is no direct correlate of protection in humans, since the studies needed to establish such a correlate in humans are challenging. For instance, protective neutralizing antibody titers have been roughly estimated for yellow fever vaccine by challenge studies in nonhuman primates and hamster models (Mason et al., 1973; Julander et al., 2011). The threshold of protective neutralizing antibody titers for SARS-CoV-2 and its lineages has not been established. Therefore, lower neutralizing antibodies titers including the samples with titers under the limit of detection as <10, do not necessarily mean susceptibility to a hypothetical secondary challenge. Samples that presented titers below 10 were not tested in lower dilutions, and if titers ≤9 are protective for each one of the different lineages of SARS-CoV-2 tested remain unknown.

It is noteworthy that the cellular immune response is believed to play an important role in the immune response for SARS-CoV-2 infection (Le Bert et al., 2020). In the present study, cellular immune response was not investigated, and only with a combined evaluation of both immune responses for a better understanding of the susceptibility to secondary infections of individuals previously infected by SARS-CoV-2. Despite concerns over the prolongation of the pandemic, the number of reinfections reported worldwide remains limited. The continuous advance of the COVID-19 vaccination has reduced the pace of new infections worldwide and is also expected to mitigate the number of reinfections as well.

The PRNT is the most specific and gold standard serological test for the differentiation of closely related flavivirus infections, as dengue and yellow fever viruses in convalescent serum samples (Calisher et al., 1989). Type-specific antibodies can be distinguished using the PRNT, and two or more flaviviruses are distinct from each other by quantitative serological criteria. Four-fold difference between PRNT titers have been used as seropositivity criterion in heterologous reactions for flaviviruses (Nemeth et al. 2007; Pauvolid-Corrêa et al. 2014). In the present study, despite some genetic and immunogenic differences observed among all lineages of SARS-CoV-2 worldwide, these viruses are closely related and for that reason a PRNT with a highly conservative threshold of 90% neutralization in ≥1:10 serum dilution was used as seropositivity criterion, as previously described (Deshpande et al., 2020).

In our group of samples, the four-fold difference among PRNT titers was not a suitable to distinguish infections among all lineages of SARS-CoV-2 tested. Four-fold or greater titers for B.1.1.28 was observed only when compared to the titers of the variants N.10, Zeta and Delta. The same was observed for B.1.1.33 titers that had four-fold higher titers only when compared to Gamma and Delta VOCs. Patients previously infected by Gamma, presented PRNT titers for Gamma more than four-fold higher for Gamma when compared to B.1 and B.1.1.28, Delta and Zeta. However, differences were smaller than four-fold when compared to B.1.1.33, N.10, and Alpha. Of note, the geometric mean of $PRNT_{90}$ titers for the Alpha variant was the second highest in all the three groups of patients, which includes samples of patients infected by B.1.1.28 and B.1.1.33 that were collected before the upsurge of Alpha variant in the United Kingdom. Despite using a highly conservative threshold, the difference in $PRNT_{90}$ titers demonstrated here among all lineages indicate that the four-fold difference as criterion of seropositivity is not reliable to distinguish lineage infections. The same is true even considering an alternative two-fold difference as criterion of seropositivity.

In conclusion, findings presented here indicate that most patients infected in early stages of COVID-19 pandemic presented neutralizing antibodies capable to neutralize wild types of all later emerged VOCs in Brazil, and that the four-fold difference in $PRNT_{90}$ titers used for other groups of viruses may not be a reliable serologic method to distinguish infection among different SARS-CoV-2 lineages in all cases.

**Acknowledgments**

We gratefully acknowledge the patients and authors from the originating laboratories responsible for obtaining the specimens. We thank the funding support from General Laboratories Coordination of Brazilian Ministry of Health; the Coordination of Health Surveillance and

Reference Laboratories of Oswaldo Cruz Foundation; Ministério da Ciência, Tecnologia, Inovações e Comunicações/Conselho Nacional de Desenvolvimento Científico e Tecnológico - CNPq/Ministério da Saúde–MS/FNDCT/SCTIE/Decit (grants no. 402457/2020-9 and 403276/2020-9); Inova Fiocruz/Fundação Oswaldo Cruz (grants no. VPPCB-007-FIO-18-2-30; FAPERJ: E26/210.196/2020). We also thank the support of the Fiocruz COVID-19 Genomic Surveillance Network (http://www.genomahcov.fiocruz.br), the Respiratory Viruses Genomic Surveillance Network of the General Laboratory Coordination, Brazilian Ministry of Health, Brazilian Central Laboratory States, the Multi-user Research Facility of Biosafety Level 3 Platform of the Oswaldo Cruz Institute and Fiocruz Diagnoses Unit Rio de Janeiro.
